# Discovery of seven novel mutations of *gyrB*, *parC* and *parE* in *Salmonella* Typhi and Paratyphi strains from Jiangsu Province of China

**DOI:** 10.1038/s41598-020-64346-0

**Published:** 2020-04-30

**Authors:** Huimin Qian, Siyun Cheng, Guoye Liu, Zhongming Tan, Chen Dong, Jinfeng Bao, Jie Hong, Dazhi Jin, Changjun Bao, Bing Gu

**Affiliations:** 1 Department of Acute Infectious Disease Prevention and Control, Jiangsu Provincial Center for Disease Prevention and Control, Nanjing, 210029 China; 2 Xuzhou Medical University School of Medical Technology, Xuzhou, 221004 China; 3 Department of Laboratory Medicine, Affiliated Hospital of Xuzhou Medical University, Xuzhou, 221002 China; 4 Centre of Laboratory Medicine, Zhejiang Provincial People Hospital, People’s Hospital of Hangzhou Medical College, Hangzhou, Zhejiang 310014 China; 5 School of Laboratory Medicine, Hangzhou Medical College, Hangzhou, Zhejiang 310053 China

**Keywords:** Antimicrobials, Bacteria, Clinical microbiology

## Abstract

Objective: To investigate the prevalence of *Salmonella* Typhi and Paratyphi resistance to quinolones and characterize the underlying mechanism in Jiangsu Province of China. Methods: Antimicrobial susceptibility testing was performed using Kirby-Bauer disc diffusion system. Quinolone resistance-determining region (QRDR), plasmid-mediated quinolone resistance (PMQR) determinant genes were detected by PCR and sequencing. Results: Out of 239 *Salmonella* isolates, 164 were *S*. Typhi and 75 were *S*. Paratyphi. 128 (53.6%) *Salmonella* isolates were resistant to nalidixic acid; 11 (4.6%) isolates to ciprofloxacin and 66 (27.6%) isolates were intermediate to ciprofloxacin. QRDR were present in 69 *S*. Typhi isolates, among which mutation at codon 83 (*n* = 45) and 133 (*n* = 61) predominated. In *S*. Paratyphi, the most common mutations were detected in *gyrA* at codon 83(*n *= 24) and *parC*: T57S (*n* = 8). Seven mutations were first reported in *Salmonella* isolates including *gyrB*: S426G, *parC*: D79G and *parE*: [S498T, E543K, V560G, I444S, Y434S]. PMQR genes including *qnrD1*, *qnrA1*, *qnrB4*, *aac (6*′*)-Ib-cr4* and *qnrS1* were detected in 1, 2, 3, 7 and 9 isolates, relatively. Conclusions: High resistance to quinolones in *Salmonella* remains a serious problem in Jiangsu, China. The presence of the novel mutations increases the complexity of quinolone-resistant genotypes and poses a threat to public health. Subject terms: *Salmonella* Typhi, *Salmonella* Paratyphi, antimicrobial resistance, QRDR, PMQR.

## Introduction

Enteric fever, including typhoid and paratyphoid fever, caused by *Salmonella enterica* serovar Typhi (*S*. Typhi) and Paratyphi (*S*. Paratyphi) A, B and C, is a global health problem. Typhoid and paratyphoid fever are transmitted primarily by the fecal-oral route and result in a variety of symptoms, including gradual onset of sustained fever, chills, nausea, rash, anorexia, abdominal pain and hepatosplenomegaly^1^. Despite the improvement in personal hygiene provision of clean water and sanitation systems, the global burden of typhoid and paratyphoid fever remains considerable. Each year, an estimated 11.9–20.6 million cases of typhoid fever occur in developing countries and cause approximately 129,000–223,000 deaths, with the majority occurring in South Asia^2^.

Antimicrobial therapy is the mainstay of treatment for enteric fever at present. Without receiving appropriate therapy, enteric fever has a case-fatality rate of 10–30%, whilst the percentage will fall to 1–4% when it is managed properly^3^. However, multi-drug resistant (MDR) *Salmonella* strains emerged and spread globally in the 1970s and 1980s due to the frequent use of traditional antimicrobials such as chloramphenicol, ampicillin and co-trimoxazole^4,5^, and since then fluoroquinolones have been regarded as the preferred drugs for the clinical treatment of typhoid fever^6^. Alarmingly, after the widespread and indiscriminate use of quinolones, cases of *Salmonella* with resistance to nalidixic acid and decreased susceptibility to fluoroquinolones have been increasingly reported in several countries^7–9^. The emergence of resistance to quinolones in *Salmonella* making the treatment of enteric fever more difficult or resulting in treatment failure.

Acquired quinolone resistance were attributable to mutations in quinolone resistance-determining region (QRDR) of DNA gyrase and topoisomerase IV, whose subunits are encoded respectively by *gyrA*, *gyrB*, *parC*, and *parE* genes^10–12^. In gram-negative bacteria such as *Salmonella*, the primary target of quinolones is *gyrA* sub-unit of DNA gyrase^13,14^. Meanwhile, plasmid-mediated resistance (PMQR) determinants *qnr* genes and *aac (6*′*)-Ib-cr4* have also been associated with resistance to quinolone^15^.

Recently, more studies have been performed to analyze the antimicrobial susceptibility and molecular characterization of *S*. Typhi and *S*. Paratyphi, but few were performed in Jiangsu Province. The objective of the present study was to evaluate the prevalence of *S*. Typhi and *S*. Paratyphi resistance to quinolones and characterize the underlying mechanism in Jiangsu, China between 2013 and 2017.

## Materials and Methods

### Specimen collection and isolate identification

This was a retrospective study of archived isolates (from stool samples) biobanked at Jiangsu Provincial Center for Disease Prevention and Control, China, between 2013 and 2017. API 20E test strips (bioMerieuxVitek, Marcy-l′Etoile, France) were used to confirm the identity of the isolates. All the isolates were then serotyped by slide agglutination with commercial antiserum (Tianrun Bio-Pharmaceutical Co., Ltd., China) according to the Kauffmann-White scheme (WHO, 2011).

### Ethical considerations

The study protocol was approved by the ethics committee of the Chinese Centre for Disease Control and Prevention (CCDC) and all experiments were performed in accordance with relevant guidelines and regulations. No informed consent was obtained from the patients because the study was retrospective. Informed consent has been waived off by the ethics committee of the Jiangsu Provincial Center for Disease Control and Prevention.

### Antimicrobial susceptibility testing

Antimicrobial susceptibility testing was performed using Kirby-Bauer disc diffusion system on Mueller-Hinton agar in compliance with the recommendations of the Clinical and Laboratory Standards Institute (CLSI) guidelines (2016) by using the following agents: ampicillin, chloramphenicol, trimethoprim-sulfamethoxazole, nalidixic acid, ofloxacin, ciprofloxacin, levofloxacin and ceftriaxone. E. coli ATCC 25922 was used as the reference strain.

### PCR amplification and DNA sequencing

The polymerase chain reaction (PCR) assays of QRDR of *gyrA, gyrB, parC* and *parE* genes were performed on the nalidixic acid-resistant (NAR) or intermediate resistant isolates. All *S*. Typhi and *S*. Paratyphi isolates were analyzed using PCR assays for the presence of PMQR determinants of *qnrA, qnrB, qnrC, qnrD, qnrS, qepA* and *aac(6*′*)-Ib-cr*. The PCR amplifications were performed using the primers shown in Table 1. Purified PCR products were sequenced by the Sanger Biotech. Sequence data were then analyzed by Bioeditor and analysed by comparison with sequences obtained from NCBI GenBank.

**Table 1:** Primers for PCR detection of antimicrobial resistance determinants.

Target	Primer sequence (5′–3′)	Annealing temperature (°C)	Amplicon size (bp)
**QRDR of Topoisomerase genes**			
*gyrA*F	TCT CCG AGA TGG CCT GAA GC	55	347
*gyrA*R	TGC CGT CAT AGT TAT CCA CG		
*gyrB*F	CAA ACT GGC GGA CTG TCA GG	55	345
*gyrB*R	TTC CGG CAT CTG ACG ATA GA		
*parC*F	CTA TGC GAT GTC AGA GCT GC	55	275
*parC*R	TGA CCG AGT TCG CTT AAC AG		
*parE*F	GAC CGA GCT GTT CCT TGT GG	60	492
*parE*R	GCG TAA CTG CAT CGG GTT CA		
**PMQR**			
*qnrA*F	GAG GAT TTC TCA CGC CAG GA	60	575
*qnrA*R	TGC CAG GCA CAG ATC TTG AC		
*qnrB1*F	GAT CGT GAA AGC CAG AAA GG	55	468
*qnrB1*R	ACG ATG CCT GGT AGT TGT CC		
*qnrB2*F	GTT GGC GAA AAA ATT GAC AGA A	57	451
*qnrB2*R	TTT GCA AGG CGT CAA ACT GG		
*qnrC*F	GGG TTG TAC ATT TAT TGA ATC	47	446
*qnrC*R	TCC ACT TTA CGA GGT TCT		
*qnrD*F	CGA GAT CAA TTT ACG GGG AAT A	60	581
*qnrD*R	AAC AAG CTG AAG CGC CTG		
*qnrS*F-1	GGA AAC CTA CAA TCA TAC ATA TCG GC	55	530
*qnrS*R-1	TAA ATT GGC ACC CTG TAG GC		
*qnrS*F-2	ATG GAA ACC TAC CGT CAC AC	55	638
*qnrS*R-2	ATA CCC AAC GCT TCG AGA AG		
*aac(6*′*)-ib-cr*F	GCA ACG CAA AAA CAA AGT TAG G	47	560
*aac(6*′*)-ib-cr*R	GTG TTT GAA CCA TGT ACA		
*qepA*F	GCA GGT CCA GCA GCG GGT AG	63	217
*qepA*R	CTT CCT GCC CGA GTA TCG TG		

## Results

### Distribution of *S*. Typhi and *S*. Paratyphi isolates

**Figure 1:**
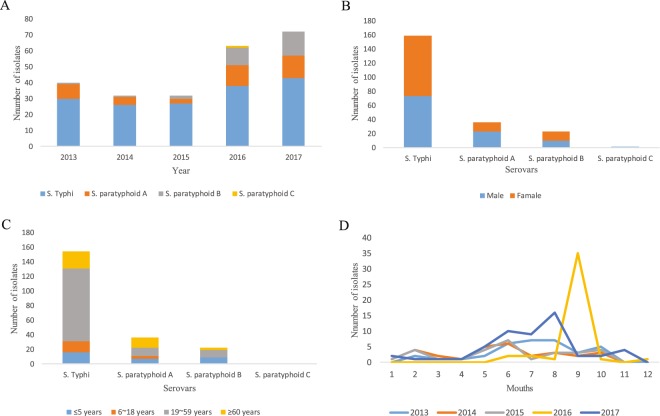
Distribution of *Salmonella enterica* serovar Typhi and *Salmonella enterica* serovar Paratyphi (**A–C**) isolates in patients by (**A**) number (**B**) gender (**C**) age and(**D**) months (2013–2017) in Jiangsu, China.

Between January 2013 to December 2017, altogether 239 *Salmonella* isolates were collected from Jiangsu, China; 106 (48.6%) were male and 112 (51.4%) were female (21 cases unknown). The age of the patients ranged from 1 month to 88 years. Among 239 *Salmonella* strains, there are 164 strains of *S*. Typhi, 44 strains of *S*. Paratyphi A, 30 strains of *S*. Paratyphi B and 1 strain of *S*. Paratyphi C. The demographic characteristics of *S*. Typhi and *S*. Paratyphi isolates are shown in Fig. 1.

During 2013–2015, enteric fever was caused mostly by *S*. Typhi (*n* = 83; 80.6%) and only 20 (19.4%) cases of *S*. Paratyphi (A and B) infection were identified. A double increase in the number of *S*. Paratyphi cases were observed with a total of 55 (40.4%) cases between 2016 and 2017, during which the number of *S*. Typhi cases remained relatively stable (Fig. 1).

### Antimicrobial susceptibility testing

As shown in Table 2, except for the 50% resistance rate to nalidixic acid, the other 10 antibacterial agents showed a relatively low resistance rate to *S*. Typhi and *S*. Paratyphi (both < 20%). The resistance to quinolones varied by subspecies. The dominating serotype resistant to nalidixic acid was *S*. Typhi (*n* = 94; 73.4%). Although only eleven *Salmonella* isolates showed resistance to ciprofloxacin (including 4 *S*. Typhi, 6 *S*. Paratyphi A and 1 *S*. Paratyphi A), intermediately resistant to ciprofloxacin were up to 66 (27.6%). The proportion of *Salmonella* isolates with susceptibility to ciprofloxacin was 100% in 2013 and down to 34.4% in 2015.

**Table 2:** Antimicrobial sensitivity patterns of *S*. Typhi and *S*. Paratyphi A, B and C isolates in Jiangsu Province, China, between 2013 and 2017.

Antimicrobial agents	*S*. Typhi (%) (N = 164)		*S*. Paratyphi(%)A (N = 44)		*S*. Paratyphi B (%) (N = 30)		*S*. Paratyphi C (%) (N = 1)		Aggregate (%) (N=239)	
	I	R	I	R	I	R	I	R	I	R
Nalidixic acid	3(1.8)	94(57.3)	1(2.3)	25(56.8)	2(6.8)	9(30.0)	0	0	6(2.5)	128(53.6)
Ciprofloxacin	58(35.4)	4(2.4)	5(11.4)	6(13.6)	2(6.8)	1(3.3)	1(100)	0	66(27.6)	11(4.6)
Ceftofur	2(1.2)	10(6.1)	0	1(2.3)	0	11(36.7)	0	1(100)	2(0.8)	23(9.7)
Cefotaxime	0	2(1.2)	0	0	1(3.3)	7(23.3)	0	0	1(0.4)	9(3.8)
Ceftazidime	1(0.6)	2(1.2)	0	1(2.3)	1(3.3)	7(23.3)	0	0	3(1.3)	9(3.8)
Ceftriaxone	1(0.6)	3(1.8)	0	0	0	7(23.3)	0	0	1(0.4)	10(4.2)
Ampicillin	0	16(9.8)	0	2(4.7)	0	11(36.7)	0	1(100)	0	30(12.6)
Amoxicillin-clavulanic acid	3(1.8)	3(1.8)	0	2(4.7)	0	11(36.7)	0	0	5(2.1)	16(6.7)
Gentamicin	1(0.6)	2(1.2)	0	0	1(3.3)	5(16.7)	0	0	2(0.8)	7(2.9)
Tetracycline	2(1.2)	5(3.0)	2(4.7)	2(4.7)	0	15(50.0)	0	1(100)	4(1.7)	23(9.6)
Sulfamethoxazole	0	2(1.2)	2(4.7)	1(2.3)	0	12(40.0)	0	1(100)	2(0.8)	16(6.7)

R- Resistant, I- Intermediate.

MDR was observed in 21 isolates of the *Salmonella*, in which 12, 8, 2 and 1 strains were found in *S*. paratyphoid B, *S*. Typhi, *S*. Paratyphi A and *S*. Paratyphi C respectively. Interestingly, both *S*. Typhi and *S*. paratyphoid A isolates were highly susceptible to gentamicin, ceftazidime, cefotaxime and amoxicillin-clavulanic acid. On the contrary, *S*. Paratyphi B was severely resistant to 9 antimicrobials except for gentamicin and ciprofloxacin (> 20%). In addition, a case of serotype Paratyphi C was detected in 2016 which was susceptible to nalidixic acid but intermediate to ciprofloxacin (Table 2).

### Identification of quinolone resistance-encoding genes in *Salmonella* Typhi

**Figure 2:**
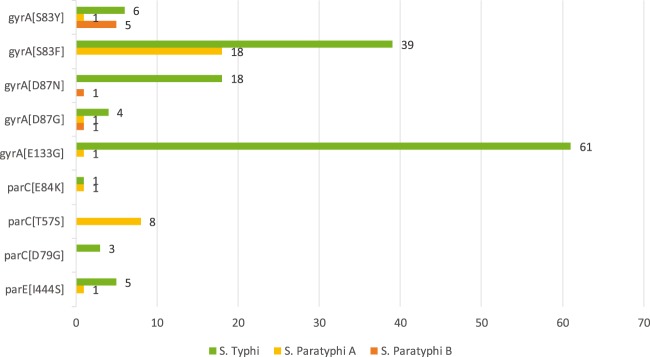
Number of different cases of major mutations among *Salmonella enterica* serovar Typhi and *Salmonella enterica* serovar Paratyphi isolates.

Number of different cases of major mutations among *Salmonella* are shown in Fig. 2. The mutations of QRDR were examined in 94 nalidixic acid–resistant *S*. Typhi strains. There were 69 (73.4%) isolates containing one or more mutations encoding at least. The most common target mutation identified was *gyrA*: E133G, accounting for 88.4%, followed by mutation at codon 83 in the *gyrA* gene (S83F/S83Y, 65.2%). 22 *S*. Typhi isolates harbored *gyrA* mutation at amino acid position 87, leading to aspartic acid replaced by asparagine or glycine. In the *gyrB*, mutation was only identified in S426G which was first reported in *S*. Typhi. Additionally, *parC* gene mutations were detected in 4 *S*. Typhi isolates including E84K (*n* = 1) and novel mutation D79G (*n* = 3). Four other novel mutations were found in *parE* in 7 isolates of *S*. Typhi (including E543K, V560G, I444S, Y434S). Particularly, all the mutations of *gyrB*, *parC* and *parE* were detected with the concomitant presence of E133G in *gyrA*. The correlation of quinolones and nucleotide changes within the QRDR is shown in Table 3.

**Table 3:** Combinations of quinolone phenotypes and genotypes identified in *Salmonella* Typhi and Salmonella Paratyphi A and B in Jiangsu, China between 2013 and 2017.

Phenotypic profile		Genetic resistance determinant		
NAL	CIP	*S*. Typhi (N = 94)	*S*. Paratyphi A (N = 25)	*S*. Paratyphi B (N=9)
R	S	WT (8)	WT (2)	WT (0)
		*qnrS1* (1)	*gyrA*: S83F (6)	*gyrA*: D87G (1)
		*qnrB4* (1)	*gyrA*: S83F; *parC*: T57S (7)	*gyrA*: D87N (1)
		*gyrA*: S83Y (1)	*gyrA*: S83F; *parE*: I444S (1)	*gyrA*: S83Y (4)
		*gyrA*: E133G (1)		*parE*: S498T (1)
		*gyrA*: D87N (2)		
		*gyrA*: D87N; *gyrA*: E133G (10)		
		*gyrA*: S83F; *gyrA*: E133G (13)		
		*gyrA*: S87G; *gyrA*: E133G (1)		
		*gyrA*: E133G; *gyrB*: S426G (1)		
		*gyrA*: D87N; *aac(6*′*)-ib-cr4* (1)		
		*gyrA*: S83F; *gyrA*: E133G; *gyrA*: D79G (3)		
Total		43	16	7
R	I	WT (15)	WT (1)	WT (0)
		*gyrA*: E133G (1)	*gyrA*: S83F (1)	*qnrA1*; *gyrA*: S83Y (1)
		*gyrA*: D87N (1)	*aac(6*′*)-ib-cr4* (1)	*qnrS1; qnrD1 aac(6*′*)-ib-cr4* (1)
		*gyrA*: S83Y (3)		
		*gyrA*: S83F; *gyrA*: E133G (18)		
		*gyrA*: D87G; *gyrA*: E133G (2)		
		*gyrA*: D87N; *gyrA*: E133G (2)		
		*gyrA*: S83F; *gyrA*: E133G; *parE*: I444S (4)		
		*gyrA*: S83F; *gyrA*: E133G; *parE*: I444S; *parE*: Y434S (1)		
Total		47	3	2
R	R	WT (1)	WT (1)	WT (0)
		*gyrA*: D87G; *gyrA*: E133G (1)	*gyrA*: S83F (2)	
		*gyrA*: D87N; *aac(6*′*)-ib-cr4* (1)	*gyrA*: S83Y (1)	
		*gyrA*: S83Y; *gyrA*: D87N; *gyrA*: E133G; *parC*: E84K (1)	*gyrA*: E133G (1)	
			*gyrA*: S83F; *gyrA*: D87G; *parC*: T57S; *parC*: E84K (1)	
Total		4	6	0

NAL, nalidixic acid; CIP, ciprofloxacin; R – Resistant; I – Intermediate; S – Susceptible; WT – Wild Type.

In addition, *qnrB4* (*n* = 1), *qnrS1* (*n* = 1) and *aac (6*′*)-Ib-cr4* (*n* = 2), which were PMQR determinants, were detected in four *S*. Typhi isolates. Two *aac (6*′*)-Ib-cr4* positive isolates coexisted with the *gyrA*: D87N mutation, one of which was MDR and resistant to ciprofloxacin.

### Identification of quinolone resistance-encoding genes in *Salmonella* Paratyphi A, B and C

Out of the 34 strains of *Salmonella* Paratyphi with resistance to nalidixic acid, high levels of mutations in QRDR were observed in *Salmonella* Paratyphi A (80%) and B (88.9%). The most common mutations in QRDR were detected in *gyrA*: S83F in *S*. paratyphoid A (*n* = 18) and *gyrA*: S83Y in *S*. paratyphoid B (*n* = 5) (Fig. 2). All eight isolates which carried *parC*: T57S mutation were *S*. Paratyphi A accompanied with the presence of S83F in *gyrA*. Among them, susceptible to ciprofloxacin was found in 7 strains, except one isolate carrying additional mutations in *gyrA*: D87N and *parC*: E84K. Only 2 point mutations were observed in *parE*, I444S (*S*. Paratyphi A) and S498T (*S*. Paratyphi B), which were novel. All *parC* and *parE* were detected with the concomitant presence of S83F in *gyrA*.

PMQR genes in *S*. Paratyphi A and *S*. Paratyphi C were only found in *aac (6*′*)-Ib-cr4* and *qnrS*1, respectively, and both of them were MDR. Among all 30 *S*. Paratyphi B isolates, 11 strains contained PMQR genes including *qnrS1* (*n* = 7), *aac (6*′*)-Ib-cr4* (*n* = 4), *qnrB4* (*n* = 2), *qnrA1* (*n* = 2) and *qnrD1* (*n* = 1). None of the isolates were positive for *qnrC* and *qepA*. The five strains carrying the *aac (6*′*)-Ib-cr4* gene were all MDR and had reduced susceptibility to quinolones. In addition, 5 strains of *S*. Paratyphi B with a single point mutation in *qnrS1* were susceptible to nalidixic acid and ciprofloxacin.

## Discussion

In this study, we explored the correlation between the level of resistance and the associated mechanism in *S*. Typhi and *S*. Paratyphi isolates obtained from Jiangsu Province of China during 2013–2017. Among the 239 isolates, 164 (68.9%) were *S*. Typhi and 75 (31.4%) were *S*. Paratyphi (including 44 *S*. Paratyphi A, 30 *S*. Paratyphi B and 1 *S*. Paratyphi C) showing a predominance of *S*. Typhi over *S*. Paratyphi, which is consistent with the previous studies^16–19^. However, the prevalence of *S*. Paratyphi collected between 2016 and 2017 is much higher than that between 2013 and 2015, showing an increasing trend and a change in species of *Salmonella* in Jiangsu Province of China. Overall, many regions in the world have already seen the increase in the prevalence of *S*. Paratyphi^16,20,21^. The reason for this change may be related to the improvements in environmental conditions and the increased use of Vi polysaccharide vaccine in recent years^22^.

The continued increase in the resistance of *Salmonella* to quinolones is a global problem, especially in Asian countries including India, Pakistan and Bangladesh^23^. The *S*. Typhi and *S*. Paratyphi isolates in this study showed a high resistance rate (53.8%) against nalidixic acid, similar to previous reports in Jiangsu Province^24^. Given the emergence of resistance to nalidixic acid for *Salmonella*, ciprofloxacin has been developed as a new quinolone and proved to be a highly effective treatment alternative. However, *Salmonella* resistance to ciprofloxacin has also become a troublesome problem due to continued abuse of quinolones in patients with diarrhea in local communities without considering the cause of diarrhea. In our study, all *Salmonella* were susceptible to ciprofloxacin in 2013, but by 2015, only 34.4% remained susceptible. In these five years, a total of 77 strains have reduced susceptibility to ciprofloxacin, which was associated with clinical failure^25^.

In the current study, we have analysed the sequence of *gyrA*, *gyrB*, *parC*, and *parE* genes in all the *Salmonella* isolates with resistant or intermediate to nalidixic acid. In general, the majority of mutations were found in three highly prevalent codons at 83, 87 and 133 in *gyrA*. Mutations at codon 83 and 87 in *Salmonella* isolates associated with reduced susceptibility to quinolones have been described previously^26–29^. Nevertheless, what noteworthy is that the most frequent mutation in *S*. Typhi was observed at codon 133 in *gyrA*, outside the usual QRDR amino acid mutations between 67 and 106. Of the 70 isolates of *S*. Typhi containing at least one mutation in QRDR, 57(81.4%) shared a common mutation in *gyrA*: E133G and only 2 isolates showed a single point mutation in *gyrA*: E133G. According to previous experiments performed in *Salmonella*, mutation at codon 133 alone could not necessarily lead to quinolone resistance, unless combination with either a second mutation in the same gene at codon 83 or 87^30–32^.

In our study, all *S*. Paratyphi A with *parC*: T57S and *gyrA*: S83F were susceptible to ciprofloxacin. The mere combination of these double mutations does not appear to reduce the susceptibility of ciprofloxacin. The data corroborated the previous demonstration that *parC*: T57S is a spontaneous compensatory mutation that makes *Salmonella* resistant to nalidixic acid but more sensitive to ciprofloxacin^33,34^. However, when additional mutation *gyrA*: D87N and *parC*: E84K were carried with mutations in *parC*: T57S and *gyrA*: S83F, it turned to resistant. Another strain harbored the mutation *parC*: E84K in *S*. Typhi also showed resistance. Accumulation of mutations at codon 83 and 87 in *gyrA* and simultaneous mutation of *parC*: E84K may be associated with full resistance to ciprofloxacin^35,36^.

Quinolone resistance in *Salmonella* is mainly mediated by mutations in *gyrA* and *parC*^19^, with few reported cases of *gyrB* and *parE*^37^. However, 8 isolates were detected containing mutations in *gyrB*: S426G, and *parE*: [S498T, E543K, V560G, I444S, Y434S] in the current study. To our knowledge, these mutations were the first reports in *Salmonella*. Interestingly, almost all *Salmonella* isolates with novel mutations were simultaneously detected common mutations in *gyrA*: [S83F and E133G], except one strain that had single E133G in *gyrA*. Five novel mutations including *gyrB*: S426G, *parC*: D79G and *parE*: [S498T, E543K, V560G and Y434S] showed susceptibility to ciprofloxacin. It showed a very strong correlation between novel mutations and mutations at codon 83 and 133. We speculate that the current mutations at 83,133 may induce the generation of other novel mutations, making the mutation genotype more complicated. However, whether these novel mutations are related to quinolone resistance remains to be confirmed by further experiments. In addition, 5 strains of *S*. Typhi with the triple mutation combination *gyrA*: [S83F, E133G] and *parE*: I444S were found intermediate to ciprofloxacin. This suggests that the novel mutation at codon 444 of *parE* gene might attenuate the activity of ciprofloxacin. The change at codon 444 could indirectly affect the combination of topoisomerase IV and fluoroquinolone, thus decreasing drug sensitivity^38^. Overall, the presence of new mutations among *S*. Typhi and *S*. Paratyphi isolates increases the complexity of quinolone-resistant genotypes and raises a critical warning to the prevalence of resistance.

Since the PMQR determinants were initially identified in 1998, various PMQR genes have been constantly detected in *Salmonella* across the world^39–41^. In this study, we found that the prevalence of PMQR genes was 7.1% in a collection of 239* S*. Typhi and *S*. Paratyphi isolates. Overall, PMQR genes were detected with lower frequencies. Among them, the *qnrS1* was the most prevalent gene, followed by *aac (6*′*)-Ib-cr4* and the *qnrB4*, *qnrA1* and *qnrD1*, which is consistent with previous reports^42–44^. PMQR genes alone in *S*. Typhi and *S*. Paratyphi could not lead to quinolone resistance, unless they co-existed with *gyrA* QRDR mutations^18^. In the current study, we found that 2 strains contained PMQR genes and mutations at codons 83 or 87 had reduced susceptibility to ciprofloxacin. Additionally, almost all *Salmonella* carrying the *aac (6* ′)*-Ib-cr4* gene described in this report were MDR, indicating that *aac (6*′)-*Ib-cr4* genes could easily lead to MDR phenotype.

Although *S*. Typhi and *S*. Paratyphi shared similarities in many aspects such as resistance and mechanisms, there are still differences between them, which is more obvious in *S*. Paratyphi B^45^. In the dataset described herein, antibiotic resistance in *S*. Paratyphi B isolates was more severe. The resistance rate of *S*. Paratyphi B to the tested antibiotics other than nalidixic acid and ciprofloxacin was 3–30 times that of *S*. Typhi. This suggests that the dispersed clone of *S*. Paratyphi B may has unique mechanisms. In the present study, PMQR genes were mainly detected in *S*. Paratyphi B, accounting for 64.7% and all *S*. Paratyphi B resistant to nalidixic acids can detect mutations at QRDR or PMQR. As a result, the ratio of MDR in *S*. Paratyphi B was about 10 times that of *S*. Paratyphi A and *S*. Typhi. This means that typhoid fever caused by *S*. Paratyphi B is more difficult to treat clinically, so typhoid and paratyphoid fever should be considered as different diseases^5,46^.

In conclusion, this study described antimicrobial resistance and the mechanisms of *S*. Typhi and *S*. Paratyphi strains isolated between 2013 and 2017 in Jiangsu Province of China. Because of the increasing prevalence of *S*. Typhi and *S*. Paratyphi isolates and indiscriminate use of antimicrobials, the rise in quinolone-resistant *Salmonella* strains and the spread of quinolone resistance-encoding genes are extremely worrying. Moreover, the emergence of novel mutations in *gyrB*, *parC* and *parE* genes increases the complexity of quinolone-resistant genotypes and poses a threat to public health. To better manage and prevent the spread of antimicrobial resistance, it is necessary to provide clinicians and local governments with accurate epidemiological information concerned. Further research and continuous dynamic monitoring of antibiotic susceptibility would be useful in the treatment and control of this infection.

## Data Availability

The datasets generated during the current study are available from the corresponding author on reasonable request.
